# ROK and Arteriolar Myogenic Tone Generation: Molecular Evidence in Health and Disease

**DOI:** 10.3389/fphar.2017.00087

**Published:** 2017-02-23

**Authors:** Ahmed F. El-Yazbi, Khaled S. Abd-Elrahman

**Affiliations:** ^1^Department of Pharmacology and Toxicology, Faculty of Medicine, American University of BeirutBeirut, Lebanon; ^2^Department of Pharmacology and Toxicology, Faculty of Pharmacy, Alexandria UniversityAlexandria, Egypt; ^3^Department of Cellular and Molecular Medicine, Faculty of Medicine, University of OttawaOttawa, ON, Canada

**Keywords:** rho-associated kinase, myogenic response, calcium sensitization, actin polymerization, integrins, type 2 diabetes

## Abstract

The myogenic response is an inherent property of resistance arteries that warrants a relatively constant blood flow in response to changes in perfusion pressure and protect delicate organs from vascular insufficiencies and excessive blood flow. This fundamental phenomenon has been extensively studied aiming to elucidate the underlying mechanisms triggering smooth muscle contraction in response to intraluminal pressure elevation, particularly, Rho-associated kinase (ROK)-mediated Ca^2+^-independent mechanisms. The size of the resistance arteries limits the capacity to examine changes in protein phosphorylation/expression levels associated with ROK signaling. A highly sensitive biochemical detection approach was beneficial in examining the role of ROK in different force generation mechanisms along the course of myogenic constriction. In this mini review, we summarize recent results showing direct evidence for the contribution of ROK in development of myogenic response at the level of mechanotransduction, myosin light chain phosphatase inhibition and dynamic actin cytoskeleton reorganization. We will also present evidence that alterations in ROK signaling could underlie the progressive loss in myogenic response in a rat model of type 2 diabetes.

## The Myogenic Response

Over 100 years ago, Bayliss (1902) elegantly demonstrated a unique property of arterioles whereby these vessels constricted in response to an increase in perfusion pressure and dilated when the internal pressure was reduced. In his study, the arterial reaction was found to be “myogenic” in nature, inherent to the vascular smooth muscle coat of the arteries, and not dependent on neuronal supply. Later studies showed that this phenomenon existed in a variety of vessel types including mesenteric, skeletal muscle, renal, and cerebral arterioles (Cole and Welsh, 2011; Davis, 2012; Abd-Elrahman et al., 2015b).

Pressure-induced tone is crucial for various aspects of the cardiovascular homeostasis. The sum of effects of endothelial activation by blood flow, sympathetic neuronal control, and humoral factors modulates the myogenic response and sets the peripheral vascular resistance and thus contributes to blood pressure regulation. Moreover, the arteriolar myogenic response tightly regulates the capillary hydrostatic pressure against fluctuations in systemic pressures thus, stabilizing capillary flow and shielding downstream structures from the damaging effects of high intravascular pressure (Davis, 2012). Thus, an abnormal myogenic response could have major consequences for various organ functions and may form the basis of vascular pathologies.

Advances in the study of myogenic tone over the last two decades unfolded the crucial role of Rho-associated kinase (ROK) in the myogenic response of resistance arteries. In this mini review, we will focus on the role of ROK in myogenic constriction in health and disease condition, specifically type 2 diabetes. We will provide a summary of our findings pertaining to the direct detection of ROK phosphorylated protein targets in the context of myogenic constriction using a highly sensitive western blotting detection technique.

## The Molecular Mechanism of the Myogenic Response

**FIGURE 1:**
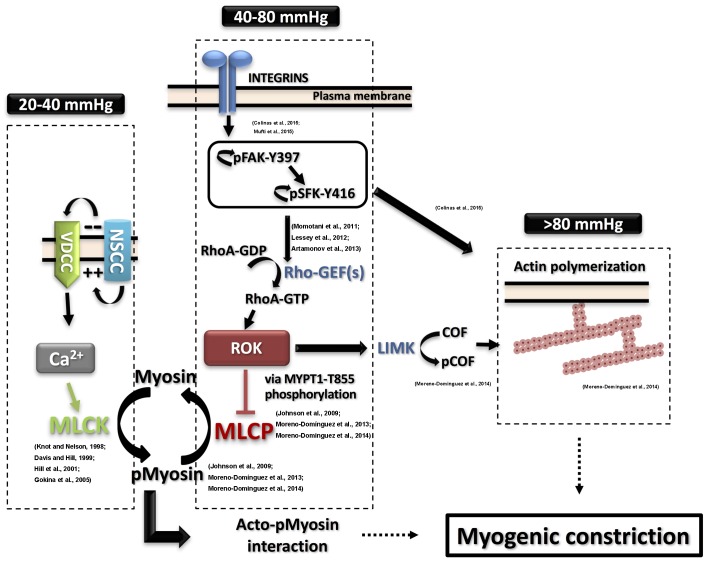
**Detailed contemporary model for the molecular basis of the myogenic response**. Intravascular pressure between 20 and 40 mmHg evokes depolarization via non-selective cation channels (NSCC), increases voltage-dependent Ca^2+^ channels (VDCC) activity, a rise in cytosolic Ca^2+^ concentration ([Ca^2+^]_i_) and vasoconstriction due to activation of myosin light chain kinase (MLCK) and phosphorylation of myosin. The resulting activation of MLCK and inhibition of myosin light chain phosphatase (MLCP) afforded by basal Rho-associated kinase (ROK) activity lead to increased pMyosin content. The release of Ca^2+^ from SR in the form of waves also contributes to the global rise of ([Ca^2+^]_i_. Intravascular pressures between 40 and 80 mmHg stimulate integrins leading to focal adhesion kinase (FAK) activation by autophosphorylation at Y397. FAK autophosphorylation permits Src family kinase (SFK) binding and its subsequent autophosphorylation at Y416 and activation. SFK in turn phosphorylates FAK to further enhance its catalytic activity, leading to activation of Rho guanine nucleotide exchange factors (RhoGEFs) to exchange GTP for GDP of RhoA. RhoA-GTP is the active form of RhoA that activates ROK to phosphorylate MYPT1-T855 and suppress MLCP activity. Further elevation of pressure (>80 mmHg) triggers actin polymerization downstream of FAK and SFK activation. ROK contributes to the control of actin dynamics via suppression of cofilin (COF)-mediated severing of actin filaments by LIM kinase (LIMK)-mediated phosphorylation of cofilin. Supporting literature is cited on the figure.

The cascade of events between the initial pressure rise and the increased force generation was subject to extensive study. Numerous studies utilized various experimental techniques to offer models and explanations of the molecular processes involved in pressure sensation and signal transduction to force-production mechanisms involving cross-bridge cycling and cytoskeletal reorganization. A widely accepted model of smooth muscle contraction involves an increase in the cytosolic calcium levels, which, via the stimulation of calcium and calmodulin-dependent kinase, activates myosin light chain kinase (MLCK) with the subsequent phosphorylation of the 20 kD regulatory light chain of myosin (LC20) leading to increased actin/myosin interaction followed by cross-bridge cycling and cell shortening (Davis and Hill, 1999; Hill et al., 2001; **Figure 1**).

### Pressure Sensing, Mechanotransduction, and Membrane Depolarization

In 1998, Knot and Nelson published an elegant study showing a strong correlation between intraluminal pressure increase, membrane depolarization, increased intracellular calcium, and vessel constriction in isolated pressurized rat cerebral arteries (Knot and Nelson, 1998). Thus, the traditional view of the initiation of myogenic contractility holds that the pressure-dependent membrane depolarization is followed by the activation of voltage-dependent calcium channels and calcium influx leading to an elevation of intracellular calcium. This pressure-dependent membrane depolarization is triggered by a pressure-sensing element existing in or interacting extensively with the smooth muscle cell membrane, that is activated by changes in intravascular pressure (Davis, 2012).

Several membrane proteins were proposed as candidates for the function of the pressure sensor; among which are integrins, stretch-activated ion channels, and G protein-coupled receptors (GPCRs). Integrins are heterodimeric transmembrane proteins that form a unique link between the smooth muscle cytoskeleton and extracellular matrix that is suitable for bi-directional signal transfer (Hill and Meininger, 2012). One accepted model for the initiation of myogenic constriction is that the increased wall tension exerted by intraluminal pressure leads to structural deformities in the extracellular matrix proteins exposing integrin-interacting motifs that initiate downstream signaling required for development of myogenic constriction (Hill and Meininger, 2012).

On the other hand, the assumption that a stretch-activated cation channels, such as transient receptor potential (TRP) channels and the epithelial sodium channels, act as a mechanosensor becomes reasonable given the fact that an early event in the cascade culminating in the arterial myogenic contractility in response to increased intraluminal pressure is smooth muscle membrane depolarization (Kizer et al., 1997; Jernigan and Drummond, 2005; Sharif-Naeini et al., 2008; Carlson and Beard, 2011; Kim et al., 2012). Yet, recent reports support a direct role for GPCRs as mechanosensors independent of activation by their respective ligands. Angiotensin receptor of the AT1 sub-type was shown to switch to an active conformation in response to cell stretch in a ligand-independent manner (Yasuda et al., 2008). The same receptor was shown later to contribute to the myogenic response of mesenteric, skeletal, and renal arterioles via coupling to Gq/11 signaling cascades (Schleifenbaum et al., 2014; Hong et al., 2016). Additionally, activation of one TRP channel (TRPM4) and its contribution to membrane depolarization and myogenic constriction was shown to be dependent on mechanical activation of G-protein coupled purinergic receptors with subsequent increase in ROK activity in response to pressure (Li et al., 2014; Li and Brayden, 2017). Another study demonstrated a clear involvement of the P2Y purinergic receptor in the development of mesenteric myogenic tone with implicated signaling to RhoA (Kauffenstein et al., 2016). Interestingly, activation of sphingosine-1-phosphate (S1P) receptors in response to pressure-induced S1P generation could be another mechanism by which a GPCR contribute to mechanotransduction (Kroetsch and Bolz, 2013; Sauve et al., 2016). This pathway was linked early on to the activation of RhoA/ROK signaling (Bolz et al., 2003). Other GPCR types were implicated but further validation is needed to support this mechanotransduction mechanism.

### Calcium-Independent Mechanisms

Several lines of evidence suggested that calcium-mediated MLCK activation and LC20 phosphorylation cannot be the only mechanism underlying force generation during the myogenic response. Studies employing ratiometric calcium dyes (Fura-2) to determine changes in the concentration of intracellular calcium in the pressure range eliciting the myogenic response demonstrated little to no change in calcium levels with increasing pressure. In 1997, D’Angelo and colleagues showed that despite the differences in the magnitude of myogenic constriction produced by different intravascular pressure levels, there were no differences detected in the steady state intracellular calcium concentration (D’Angelo et al., 1997). In 2002, Osol and colleagues demonstrated similar results whereby membrane depolarization and intracellular calcium concentration did not change with increased force generation in the pressure range associated with myogenic constriction (60–140 mm Hg) (Osol et al., 2002). These and many other observations (VanBavel et al., 2001; Lagaud et al., 2002; Bolz et al., 2003; Gokina et al., 2005) support the involvement of calcium-independent mechanisms in the development of myogenic contractility. Studies demonstrated an increased sensitivity of tone production to intracellular calcium with increased intravascular pressure making an early case for calcium-sensitization to be among these mechanisms (VanBavel et al., 1998; Schubert et al., 2002). In a physiological context where resistance arterioles are continuously producing force in the face of maintained intravascular pressure, a contractile mechanism that does not involve massive fluctuations of calcium would afford the advantage of conserving calcium signaling given the ubiquitous nature of calcium-mediated processes (Schubert et al., 2008).

### The Role of ROK

As mentioned earlier, not only was ROK reported to initiate smooth muscle depolarization during myogenic constriction (Li and Brayden, 2017), it was also reported to exert a modulatory effect on depolarization by inhibiting delayed rectifier potassium channels (Luykenaar et al., 2009). However, ROK-mediated calcium sensitization was implicated as an underlying mechanism in many of the studies cited above in support of calcium-independent mechanisms. Calcium sensitization refers to a mechanism whereby myosin light chain phosphatase (MLCP) inhibition shifts the equilibrium toward the accumulation of phosphorylated LC20 without an associated increase in calcium-induced MLCK activity (Somlyo and Somlyo, 2003). ROK-dependent phosphorylation of the myosin targeting subunit of MLCP (MYPT1) was reported to occur at two threonine residues, T697 (rat numbering) reported to inhibit MLCP catalytic activity, and T855 (rat numbering) reported to inhibit both the catalytic activity and myosin binding (Muranyi et al., 2005). Another potential pathway for calcium sensitization involves the protein kinase C (PKC)-mediated phosphorylation of a 17 kD protein phosphatase inhibitor (CPI17) increasing its inhibitory effect of MLCP (Hayashi et al., 2001). So far, a pressure-induced change in CPI17 phosphorylation was not detected in the context of myogenic response (Johnson et al., 2009). However, the involvement of other PKC-mediated mechanisms remains a very likely possibility (Lagaud et al., 2002; Johnson et al., 2009; Moreno-Domínguez et al., 2014; El-Yazbi et al., 2015; Hong et al., 2016).

On the other hand, an additional mechanism that can be involved in potentially calcium- and LC20 phosphorylation-independent manner is actin cytoskeleton reorganization. Relatively recent evidence suggested that actin cytoskeleton is not a static structure in the context of generating and transmitting force in smooth muscle (Gunst and Zhang, 2008). The role of actin cytoskeleton polymerization was implicated in myogenic constriction as early as 1998 with the observation that treatment with cytochalasin D, an agent that disrupted actin cytoskeleton, caused forced dilation of pressurized arteries to occur at lower pressures (Cipolla and Osol, 1998). An early report by Cipolla et al. (2002) provided quantitative evidence to support *de novo* actin polymerization in response to pressure increases. This was later supported by qualitative evidence showing that F-actin content increased in response to intraluminal pressure elevation (Flavahan et al., 2005). Both studies showed that cytochalasin D inhibited both actin polymerization and the myogenic response. A role for Rho A/ROK-mediated signaling was also implicated in smooth muscle actin cytoskeletal dynamics involved in contraction and force generation (Numaguchi et al., 1999; Gerthoffer, 2005).

A third calcium-independent mechanism that could be associated with smooth muscle contraction is thin filament regulation (Morgan and Gangopadhyay, 2001). Studies of the myogenic constriction of rat cerebral arterioles, ruled out the contribution of this mechanism based on a lack of detection of an increased calponin and caldesmon phosphorylation (Moreno-Domínguez et al., 2014). These experiments were conducted using a western blotting method of high sensitivity (∼200-fold) that offered an exclusive approach to accurately quantify increases in protein phosphorylation in minute tissue segments such as those used in isobaric myography experiments (Takeya et al., 2008). Prior to this technical advance, direct quantification of ROK activity and assessment of its contribution to myogenic constriction of resistance arteries was a daunting task (Schubert et al., 2002, 2008). This biochemical approach was employed in a series of studies to track changes in protein phosphorylation in different mechanotransduction and force generation pathways leading to myogenic constriction of cerebral and skeletal muscle arterioles as outlined below, providing a molecular context for the role of ROK in these processes in vessels from healthy and type 2 diabetic rat model.

## ROK-Mediated Calcium Sensitization in Pressure-Induced Myogenic Contractility

A main limitation of using pharmacological inhibitors of ROK to assess the presumed contribution of this pathway in the myogenic tone is the lack of differentiation between whether the observed tone reduction is due to suppression of basal (pressure-independent) ROK-mediated sensitization vs. inhibition of a pressure-evoked ROK activity (Schubert et al., 2008). Several elegant experimental designs were employed to circumvent this limitation including the assessment of the relative sensitivity of the intracellular calcium vs. tone relationship to ROK inhibitors at different pressure levels (Schubert et al., 2002), the demonstration of the need for a higher concentration of the inhibitor compound to obtain the same level of attenuation of tone at higher pressures (Gokina et al., 2005), and increased Rho A translocation to cell membrane and presumed ROK activation in response to increased intravascular pressure (Dubroca et al., 2007).

A direct link of the perceived pressure-evoked ROK activation to a downstream effector was shown by examining the hypothesis that increased intravascular pressure leads to a corresponding graded increase in ROK-mediated MYPT1 phosphorylation, MLCP inhibition, and increased LC20 phosphorylation (Johnson et al., 2009; Moreno-Domínguez et al., 2013). These findings are summarized in **Figure 1**. Using the high-sensitivity three-step detection method, a graded increase in MYPT1 phosphorylation was evident. These findings implicated MYPT1 phosphorylation at T855 as the more likely site for regulation of MLCP activity in the context of pressure-induced contraction whereby myogenic constriction associated with step increases in intraluminal pressure from 20 mmHg to 60 and 100 mmHg was associated with an increase only in T855 phosphorylation. A corresponding gradual increase in LC20 phosphorylation was also observed and both increases were sensitive to pharmacological inhibition of ROK. No change in T697 phosphorylation was recorded in the pressure range of 60–120 mmHg and this observation was consistent in both rat cerebral and skeletal muscle arterioles. Interestingly, vessels segments from these vascular beds showed significant basal (low pressure) levels of MYPT1 phosphorylation at both sites, with only T855 phosphorylation being sensitive to ROK inhibition (Johnson et al., 2009; El-Yazbi et al., 2010). Abolition of basal MYPT1 T855 phosphorylation at low pressure by ROK inhibition prevented myogenic tone development (Johnson et al., 2009). These results provide direct evidence for the need of ROK-mediated sensitization in both basal and pressure-evoked states.

Thus, the latter observation triggered a question of whether previous data reporting the involvement of ROK-dependent calcium sensitization via MYPT1 phosphorylation in agonist-induced microvascular constriction pertains to basal vs. evoked increased in ROK-mediated MYPT1 phosphorylation. Studies using serotonin as a model vasoconstrictor agonist in the cerebral circulation produced variable results. Sandoval et al. (2007) concluded that serotonin treatment increased ROK-dependent myofilament calcium sensitivity in ovine cerebral arteries. Yet another study demonstrating the sensitivity of the serotonin-evoked tone to ROK inhibitors was not able to demonstrate an increase in MYPT1 phosphorylation even in the larger rabbit basilar artery segments under control conditions (Watanabe et al., 2005). Using the highly sensitive biochemical detection method, serotonin evoked constriction and increased LC20 phosphorylation at low pressure levels associated with basal MYPT1 phosphorylation, though completely abolished by ROK inhibition, were not associated with a further increase in MYPT1 phosphorylation (El-Yazbi et al., 2010) reflecting a need for basal rather than evoked ROK-mediated MLCP inhibition. Cerebral vessel segments allowed to constrict in response to increased intravascular pressure prior to treatment with serotonin responded by increased constriction and demonstrated a different pattern of MYPT1 phosphorylation. Serotonin constriction evoked an increase in T855 phosphorylation that was dependent on ROK activity (El-Yazbi et al., 2010). Prior studies demonstrated an interplay between pressure- and agonist-induced constriction whereby increased intraluminal pressure increases the vessel sensitivity to agonist-induced constriction (Harder, 1988; Lombard et al., 1990; VanBavel and Mulvany, 1994). These results provided the first direct evidence that this increased sensitivity occurs due to the recruitment of ROK-mediated MYPT1 phosphorylation. In a physiological milieu where the microvasculature is exposed to pressure, it is likely that increased ROK activity is the mechanism underlying the agonist mediated modulation of vascular tone. The recruitment of this pathway in response to other neurotransmitter and humoral mediators remains to be confirmed in other vascular beds and with other agonists. It remains very likely as demonstrated recently for P2Y6-mediated purinergic constriction in mouse mesenteric arterioles (Kauffenstein et al., 2016). Given the known modulation of the arteriolar myogenic response by tissue metabolic demands (Davis, 2012), it could be plausible that other physiological parameters such as temperature, tissue oxygenation level, and pH would affect ROK activity and impose an additional layer of interplay. A potential molecular link would be similar to that observed in cold-induced vasospasm whereby an enhanced ROK activity was triggered by increased mitochondrial production of reactive oxygen species culminating in vasoconstriction in response to cold temperatures (Bailey et al., 2005). Indeed, a role was suggested for reactive oxygen species in modulating calcium sensitization during the myogenic constriction in response to endogenous physiological/pathological stimuli (Nowicki et al., 2001; Veerareddy et al., 2004; Keller et al., 2006; Gebremedhin et al., 2014).

Further experimentation with combined agonist- and pressure-evoked constriction revealed situations where cerebral or skeletal muscle arterioles produced profound constrictions with no recorded increased in LC20 phosphorylation level (Moreno-Domínguez et al., 2013, 2014). With no detected increases in the phosphorylation of caldesmon and calponin, the proteins involved in thin-filament regulation, the contribution of dynamic actin cytoskeleton re-organization was examined. In addition to early studies mentioned before, the role of actin polymerization was further emphasized by observations that agents blocking actin polymerization interfered with myogenic tone generation while those increasing F-actin formation increased myogenic constriction (Cipolla et al., 2002). Yet interpretation of these results begged the question of whether myogenic contractility required an intact actin cytoskeletal framework that is well-formed basally vs. a situation where *de novo* actin polymerization occurs in response to increased intravascular pressure. Similar to previous results (Cipolla et al., 2002), a reduction in G-actin (unpolymerized) during generation of myogenic contractility was evident, this reduction was blocked with latrunculin B in rat cerebral and skeletal arterioles (Moreno-Domínguez et al., 2013, 2014). Extending the range of analysis further to higher pressure values, no further increase in LC20 phosphorylation was observed beyond 80–100 mm Hg, however, further constriction could be recorded in the face of higher pressures. Under these experimental conditions, the only biochemical parameter changing was a reduction in the G-actin pool indicating actin cytoskeleton reorganization as the sole force generation mechanism (Moreno-Domínguez et al., 2014). Analysis of the circumferential wall stress of cerebral arteriolar segments under these conditions estimated that actin polymerization accounted for ∼30% of the generated force. Both the pressure-evoked constriction and G-actin pool reduction under these conditions were sensitive to ROK inhibition. ROK appears to cause an indirect increase in the phosphorylation of cofilin through the activation of LIM kinase (Bernard, 2007). Phosphorylation suppresses cofilin ability to cap and sever actin filaments (Carlier et al., 1997; Bernard, 2007). Cofilin phosphorylation was increased with pressure-evoked constriction and G-actin reduction, an observation that was reversed in presence of a ROK inhibitor (Moreno-Domínguez et al., 2014).

Taken together, these findings provide a direct biomolecular context for the role of ROK in generation of myogenic constriction. A basal component of ROK activity appears to be required for force generation followed by a pressure-evoked increase. ROK mediates force generation in the face of increased intravascular pressure through facilitation of a spectrum of mechanisms. Initially, the maintenance of a background inhibition of MLCP allows a calcium-triggered increase in LC20 phosphorylation. A graded increase in force generation in the face of pressure is achieved by increased MYPT1 phosphorylation leading to increased LC20 phosphorylation in an intracellular environment with no appreciable increases in calcium. Meanwhile, basal ROK activity is required for the preservation of the integrity actin cytoskeleton allowing for force transmission and contraction. Yet, dynamic ROK-mediated actin reorganization appears to contribute to force generation and eventually becomes the only mechanism underlying force production after LC20 phosphorylation reaches its maximum levels.

## Mechanotransduction to ROK

Mechanosensing mechanisms involving the contribution of ion channels offer a straightforward model for linking pressure sensation to arterial constriction that is dependent on calcium-mediated MLCK activation and LC20 phosphorylation. However, models involving membrane depolarization do not provide an explanation for ROK activation. Previous reports in other cell types linked mechanical activation of integrins to increased ROK activity together with actin cytoskeleton remodeling (DeMali et al., 2003; Legate et al., 2009). Blocking integrin function using a function blocking antibody against α_5_ integrin or a peptide that selectively blocks the function of α_5_ integrin reversed the constriction obtained by increased intraluminal pressure and reduced MYPT1 T855 and LC20 phosphorylation confirming integrin signaling to ROK and MLCK/MLCP equilibrium in general (Colinas et al., 2015; Mufti et al., 2015). Moreover, vasodilation following antibody treatment was associated with actin depolymerization and increased G-actin pool, again establishing a clear connection between integrin activation and actin polymerization (Colinas et al., 2015).

Upon examination of the phosphorylation status of focal adhesion proteins in vessel segments producing myogenic constriction, increased phosphorylation of both focal adhesion kinase (FAK) at Y397 and Src family kinase (SFK) at Y416 was identified as an early and essential event for the development of myogenic constriction (Colinas et al., 2015). A pressure-dependent increase in FAK phosphorylation at Y576/577 was seen indicating a graded activity in response to pressure. Treatment with inhibitors of these kinases abolished the myogenic contractility and reduced MYPT1 and LC20 phosphorylation and actin cytoskeleton polymerization indicating that FAK and SFK are activated down stream of integrins in a pressure-dependent manner and relay signals to pathways leading to calcium sensitization and actin cytoskeleton polymerization involving ROK activity. FAK and SFK phosphorylation and activation are proposed to mediate this effect via stimulation of RhoGEFs (Momotani et al., 2011; Lessey et al., 2012; Artamonov et al., 2013). A potential pathway is outlined in **Figure 1**.

Several issues remain to be clarified by future research. The calcium dependence of the integrin-mediated FAK and SFK signaling needs to be examined. This might explain the obligate dependence of the myogenic response on calcium despite the demonstrated involvement of ROK-mediated calcium-independent pathways. In addition, several observations indicate that different force generation mechanisms are not necessarily simultaneously activated at all pressure ranges, though all appear to involve ROK activity. Whether this effect is due to a limited effective range, e.g., LC20 phosphorylation reaching a maximum level, or selective activation by disparate mechanotransduction pathways remains to be determined. As well, GPCRs remain a viable alternative pathway for mechanotransduction to ROK in need for validation with direct molecular evidence in the context of myogenic constriction.

## Alterations in ROK Activity in Pathological Conditions

Given the crucial physiological role of the myogenic response in cardiovascular homeostasis, it is predictable that dysfunctional myogenic regulation of arterial diameter will be associated with multiple vascular pathologies. Specifically, enhanced basal myogenic tone may preclude the vasodilatory response of resistance arteries in periods of high metabolic activity and oxygen demand. The reduced arterial diameter would be expected to restrict blood flow to the organ increasing the risk of ischemia. These microvascular changes could provide the starting point for further more permanent alterations in the micro/macro-vasculature (Jax, 2010). Abnormal myogenic constriction was observed in few pathological conditions such as hypertension, hemorrhagic and ischemic stroke, diabetes, and Alzheimer’s disease (Dunn et al., 1998; Smeda and King, 2000; Coulson et al., 2002; Niwa et al., 2002; Izzard et al., 2003). With ROK activity being an essential tenet of the myogenic response, it becomes an attractive target for study and for potential therapeutic interventions. Although the main focus of the next section is type 2 diabetes, we speculate that aberrations in ROK signaling leading to abnormal myogenic response in other disease conditions will be comparable to what is reported in diabetes.

**FIGURE 2:**
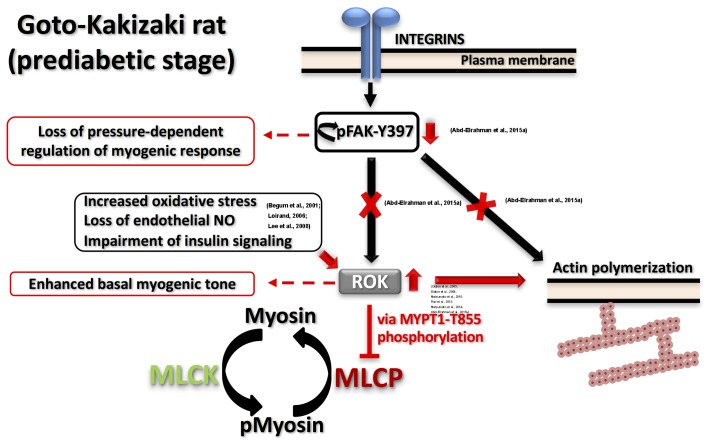
**Proposed model for dysfunctional myogenic response in prediabetic GK rats**. Two major defects were characterized in prediabetic GK cerebral arteries: (A) Augmented ROK activity at low intraluminal pressure causing: (i) higher MYPT1-T855 phosphorylation, more inhibition of MLCP and higher myosin phosphorylation; (ii) enhanced actin polymerization. (B) Abnormal FAK-Y397 phosphorylation downstream of integrin activation leading to loss of pressure-dependent regulation of myogenic response. Supporting literature is cited on the figure.

Both enhanced and reduced myogenic constriction has been described in various animal models and patients of type 2 diabetes (Lagaud et al., 2001; Schofield et al., 2002; Jarajapu et al., 2008; Kold-Petersen et al., 2012; Sauve et al., 2016). One potential cause for the apparent discrepancy in observations could be studying vascular function at different disease stages even within the same model. Goto-Kakizaki (GK) rat is a lean, polygenic origin, spontaneous onset model of type 2 diabetes that offers an exclusive opportunity to study diabetes-related characteristics without the confounding influence of hyperlipidemia or obesity (Nie et al., 2011). These rats demonstrate a phenotype of insulin resistance prior to the development of type 2 diabetes (Gao et al., 2010). Although reduced myogenic constriction was reported in GK rats (Kold-Petersen et al., 2012), longitudinal studies in cerebral arterioles of this model provided the molecular basis for the progressive loss of myogenic response from the prediabetic to the diabetic stage. Cerebral arterioles from these rats demonstrated an enhanced myogenic behavior during the prediabetic phase (Abd-Elrahman et al., 2015a). Specifically, enhanced LC20 phosphorylation and increased actin polymerization (lower G-actin level) occurred at low intraluminal pressure due to an increase in ROK activity rather than ROK protein expression. Significantly, a loss of the pressure-dependent activation of myogenic response in prediabetic GK rats could be attributed to the observed loss of pressure-dependence of FAK-Y397 phosphorylation downstream of integrin signaling. These defects in mechanotransduction might lead to an abnormal association between pressure and RhoA/ROK activation and, thus, contribute to the loss of pressure-evoked change in LC20 and G-actin contents. The proposed model of the myogenic abnormality seen in GK rats is depicted in **Figure 2**. Enhanced ROK activity rather than its level of expression was a common defect that has been characterized in most of the vascular beds of prediabetic and diabetic animal models exhibiting augmented contractile responses to agonists (Didion et al., 2005, 2006; Matsumoto et al., 2010, 2014; Rao et al., 2013). The mechanism(s) responsible for enhanced ROK activity in vascular smooth muscle cells (VSMC) of type 2 diabetic animal models has not been clearly characterized; however, a few candidates have been suggested including: (i) increased oxidative stress that stimulates the kinase activity; (ii) loss of endothelium-derived NO that normally functions to suppress ROK activity (Loirand, 2006); and/or (iii) selective impairment of insulin- and insulin receptor-mediated signaling that suppresses RhoA activation and ROK activity in VSMCs (Begum et al., 2001; Lee et al., 2008).

These observations make a case for targeting ROK as a potential therapeutic intervention for vascular disorders associated with diabetes. Indeed, Fasudil, a ROK inhibitor, is approved in Japan for the treatment of vasospasm after subarachnoid hemorrhage and also clinically effective for the treatment of pulmonary hypertension (Shi and Wei, 2013). Multiple limited clinical trials report a beneficial effect for ROK inhibitors in systemic hypertension, vasospastic angina, stable angina, ischemic stroke, and right ventricular failure (Shimizu and Liao, 2016). However, given the crucial importance of ROK in general vascular function, the utility and safety of such pharmacological interventions in treatment of the widespread vascular complications associated with diseases such as diabetes will require substantial additional investigation. Examination of the myogenic reactivity and the involvement of enhanced ROK activity in other animal models of type 2 diabetes is necessary. The high fat/high fructose fed rat provides a well-characterized model for this purpose with a slow conversion from prediabetes to diabetes (Lozano et al., 2016) and an adequately reported profile of changes in adipose inflammatory mediators (Sousa-Pinto et al., 2016). Moreover, emerging clinical evidence paints a picture whereby newer oral hypoglycemic medications might afford a direct effect on cardiovascular function leading to reduced cardiovascular risk (Zinman et al., 2015; Marso et al., 2016). Establishing whether these molecules affect vascular function, particularly myogenic contractility, and whether they can modify ROK activity and/or expression could offer valuable insight into the timing and pattern of use of these medications.

In conclusion, three major mechanisms have been well characterized to underlie myogenic constriction including (i) Ca^2+^-dependent activation of MLCK; (ii) ROK-dependent inhibition of MLCP; and (iii) ROK dependent dynamic actin cytoskeleton reorganization. It is now evident that the relative contribution of the three mechanisms during myogenic constriction is contingent on the intravascular pressure sensed by the mechanosensors in the vascular wall, yet all three share an obligate reliance on ROK activity. Moreover, the observed dysfunctional myogenic response in a type 2 diabetic rat model appeared to reflect an altered ROK activity. Delineating these alterations in type 2 diabetes and other vascular disorders will lead the search for new targets that could be clinically exploited in the future to alleviate the progression of cardiovascular diseases.

## Author Contributions

AE-Y and KA-E have participated in reviewing the literature, summarizing the results, and writing the manuscript.

## Conflict of Interest Statement

The authors declare that the research was conducted in the absence of any commercial or financial relationships that could be construed as a potential conflict of interest.
